# Web content topic modeling using LDA and HTML tags

**DOI:** 10.7717/peerj-cs.1459

**Published:** 2023-07-11

**Authors:** Hamza H.M. Altarturi, Muntadher Saadoon, Nor Badrul Anuar

**Affiliations:** 1 Department of Computer System and Technology, Faculty of Computer Science and Information Technology, Universiti Malaya, Kuala Lumpur, Kuala Lumpur, Malaysia; 2 Department of Software Engineering, Faculty of Computer Science and Information Technology, Universiti Malaya, Kuala Lumpur, Kuala Lumpur, Malaysia

**Keywords:** HTML topic model, HTM, Topic modeling, Topic models comparison, LDA, HTML tags, Web content mining, Web topic modeling, Generative model

## Abstract

An immense volume of digital documents exists online and offline with content that can offer useful information and insights. Utilizing topic modeling enhances the analysis and understanding of digital documents. Topic modeling discovers latent semantic structures or topics within a set of digital textual documents. The Internet of Things, Blockchain, recommender system, and search engine optimization applications use topic modeling to handle data mining tasks, such as classification and clustering. The usefulness of topic models depends on the quality of resulting term patterns and topics with high quality. Topic coherence is the standard metric to measure the quality of topic models. Previous studies build topic models to generally work on conventional documents, and they are insufficient and underperform when applied to web content data due to differences in the structure of the conventional and HTML documents. Neglecting the unique structure of web content leads to missing otherwise coherent topics and, therefore, low topic quality. This study aims to propose an innovative topic model to learn coherence topics in web content data. We present the HTML Topic Model (HTM), a web content topic model that takes into consideration the HTML tags to understand the structure of web pages. We conducted two series of experiments to demonstrate the limitations of the existing topic models and examine the topic coherence of the HTM against the widely used Latent Dirichlet Allocation (LDA) model and its variants, namely the Correlated Topic Model, the Dirichlet Multinomial Regression, the Hierarchical Dirichlet Process, the Hierarchical Latent Dirichlet Allocation, the pseudo-document based Topic Model, and the Supervised Latent Dirichlet Allocation models. The first experiment demonstrates the limitations of the existing topic models when applied to web content data and, therefore, the essential need for a web content topic model. When applied to web data, the overall performance dropped an average of five times and, in some cases, up to approximately 20 times lower than when applied to conventional data. The second experiment then evaluates the effectiveness of the HTM model in discovering topics and term patterns of web content data. The HTM model achieved an overall 35% improvement in topic coherence compared to the LDA.

## Introduction

Topic modeling aims to discover topics within a collection of digital textual documents. Several fields, such as the Internet of Things, Blockchain, recommender systems, software engineering, digital humanities, and political science, apply topic modeling to efficiently detect topics in large collections of data (Chung et al., 2019; Guo et al., 2018; Liu et al., 2018; Mulunda, Wagacha & Muchemi, 2018). Applying such models benefits web content mining and its several applications, such as search engine optimization (SEO), web services, web filtering, and digital marketing (Boyd-Graber, Hu & Mimno, 2017). Modeling topics in web content is a crucial and remarkable research problem in natural language processing. The usefulness of topic models depends on the quality of resulting term patterns and topics with high quality. Topic coherence is the standard metric to measure the quality of the existing topic models.

Existing topic models have three types of design: a specific design, a generic design, and an integrative design. The specific topic model works best on what they have been designed for, such as Twitter (Wang & Maeda, 2019), news (Yang, Li & Zhao, 2019), reviews (Chehal, Gupta & Gulati, 2020; Park & Liu, 2020), and healthcare (Rijcken et al., 2022). However, there is a lack of study on web content topic modeling. The generic design of the topic model works best on documents with conventional structures, such as articles, reports, and press releases. The integrative design of the topic model combines the generic topic models with clustering methods in order to cluster documents (Costa & Ortale, 2020) and XML (Costa & Ortale, 2019). This design merely uses the generic model, which makes them unsuitable for web content, as this study demonstrates in the first experiment. The Latent Dirichlet Allocation (LDA) and its variants are prominent models in the field of topic modeling due to their outstanding performance on conventional documents. Research literature applies several variants of the LDA for modeling topics in web content. These models are the Correlated Topic Model (CTM) (Blei & Lafferty, 2006), Dirichlet Multinomial Regression (DMR) (Mimno & McCallum, 2008), Hierarchical LDA (HLDA) (Teh et al., 2006), Latent Dirichlet Allocation (LDA) (Blei, Ng & Jordan, 2003), Pseudo-document based Topic Model (PTM) (Zuo et al., 2016), and Supervised Latent Dirichlet Allocation (Mcauliffe & Blei, 2008). However, these topic models are insufficient and underperform when applied to web content data and fail the coherence test. The limitation to learning the topics is due to differences in the structure of the conventional and HTML documents. Neglecting the unique structure of web content leads to missing the otherwise coherence topics and, therefore, low quality of topics. The failure of topic modeling with web content data has yet to be demonstrated and addressed in the research literature. These drawbacks raise the need to modify the current topic models to learn topics in web content data.

This study aims to propose an innovative topic model to learn topics in web content data. We present the HTML Topic Model (HTM) that takes into consideration the HTML tags to understand the structure of web pages. HTML tags, such as <title>, <metadata>, <b>, <ul>, <li>, <hr>, and <img>, contain very short textual contents. Unlike conventional text documents, combining textual contents from HTML tags results in a sparse and incoherent pattern (Figueiredo et al., 2013). The HTM employs probabilistic modeling to examine web content structures; a webpage is a set of tags. Each tag is a mixture of topics, and each detected term is assigned to a particular topic. The main contribution of this article is four-fold. We first compare and evaluate the benchmark topic models on conventional document data. Secondly, we compare and evaluate these models based on the web content data. We then demonstrate the drawbacks of each topic model by comparing their performance on both data sources. Lastly, we propose the HTM model for learning coherence topics in web content data and demonstrate the performance enhancement made by the HTM model compared to the well-known LDA.

This study has four research questions: Do the benchmark topic models perform well and generate coherent topics when applied on web content data? Which of the benchmark model have steady performance when applied to web content data? Does considering HTML tags increase the quality of the generated topics applied on web content data? Does the proposed HTM model have better metrics values than the other topic models? In order to answer these research questions, we conducted two experiments. For both experiments, we used topic coherence for performance evaluation. Topic coherence measures the consistency and quality of each individually generated topic. The first experiment examines the performance of the benchmark topic models when working with the web content data and highlights their underperformance. The results indicate the essential need for a web content topic model to generate more coherent topics for web pages. When applied to web data, the overall performance dropped up to approximately 19 times lower than when applied to conventional data. The result of this experiment answers the first and second research questions of this study. The second experiment evaluates the enhancements made by the HTM in discovering topics and term patterns of web content data. The HTM yields more meaningful topics given a number of metrics and achieved an overall 36.7% improvement in topic coherence compared to the LDA. The result of this experiment answers the third and fourth research questions of this study.

The organization of the article is as follows. Section 2 follows with a description of the existing topic models. Section 3 describes the proposed HTML topic model (HTM). The methodology and results of evaluating the existing topic models and the proposed HTM are presented in Section 4 and 5. Section 6 concludes and summarizes the article.

## Background

An immense volume of hypertext and digital documents exist online and offline with content that can offer useful information and insights. Analyzing and understanding the collection of such enormous content, however, is nearly impossible for an individual with traditional methods. Topic modeling, a text mining tool, aims to discover latent semantic structures or topics within a set of textual digital documents. Topic models are widely applied on the Internet of Things, Blockchain, chatbots, bioinformatics, recommender system, spam filtering, summarization, sentiment analysis, text categorization, text similarity, service matchmaking, and classification (Chung et al., 2019; Guo et al., 2018; Liu et al., 2018; Mulunda, Wagacha & Muchemi, 2018).

Researchers designed several topic models, some of which are generic and comprehensive, such as the LDA and the HLDA. Other models, in contrast, are designed to work with particular topics or specific tasks, such as the Author-Topic Model and the Twitter-LDA. Despite design differences and applications, topic models are Vector Space Model (VSM) based. This section categorizes the VSM topic models based on their underlying statistical model, as Fig. S1 illustrates. The following subsections address the key concepts of topic modeling and the related work of web topic modeling.

### Key concepts

The Latent Semantic Indexing (LSI) model was the first statistical model for grouping co-occurrence terms in documents. Hofmann (1999) proposed a Probabilistic Latent Semantic Indexing (pLSI), an offshoot of LSA, to improve topic modeling (Vayansky & Kumar, 2020). The pLSI combines latent semantics with a probability model to enhance the detection of the co-occurrence of topics. Blei, Ng & Jordan (2003) proposed the Latent Dirichlet Allocation (LDA), which extended the pLSI by adding Dirichlet priors on topic distributions (Alkhodair et al., 2018; Hajjem & Latiri, 2017). Several studies proposed topic models that add constraints on the traditional LDA to generate modified models called LDA variants (Chen, Thomas & Hassan, 2015). In addition to Semantic Indexing and Dirichlet Indexing, researchers use a few other computational methods for topic modeling, such as the independent component analysis (ICA) and principal component analysis (PCA). These methods exhibit efficiency in topic modeling but are still outweighed by the performance of the LDA model.

The LDA and its variants are prominent models in the field of topic modeling due to their outstanding performance. Several LDA variants exist for specific tasks, such as modeling tweets (Wang & Maeda, 2019), web services, authors (Alrabaee, Debbabi & Wang, 2020), news (Yang, Li & Zhao, 2019), social networks (Liu & Hu, 2021), and recommendations (Tang, Zhang & Niu, 2020). Although these models perform well when applied to the tasks for which they are designed, they notably underperform when applied to other tasks, such as modeling web content data.

Generic variant models of the LDA have also been proposed and applied in various tasks and applications. A few studies address the application of the LDA or its variant models on web content data (Gu et al., 2016; Liu & Forss, 2015b). Those use various techniques such as the Correlated Topic Model (CTM), the Dirichlet Multinomial Regression (DMR), Hierarchical LDA (HLDA), the Pseudo-document based Topic Model (PTM), and the Supervised Latent Dirichlet Allocation (sLDA). Our study uses these models for benchmarking due to their solid baselines and wide use. Table S1 summarizes the characteristics and limitations of these benchmark models.

It is worth mentioning that several other models, such as BERTopic and word-embedding models, are somewhat related to the benchmark models of this study. However, several key factors support excluding them from this study. The BERTopic model relies on pre-trained Bidirectional Encoder Representations from Transformers (BERT) and employs a unique strategy for word representation. This study focuses on modelling topic distribution with LDA and its variants, whereas BERTopic captures contextual semantics. The fundamental differences between mathematical foundations and topic representation methods preclude them from our experiments. Moreover, despite their effectiveness in clustering words, word embeddings are not explicitly designed for topic identification. This study seeks to propose a novel topic model for the analysis of coherent web content while highlighting the limitations of established benchmark models. By adhering to our research objectives, we provide valuable insight into the need for innovative topic modelling techniques in the field of web mining.

### Related work

Web mining is discovering and extracting information and knowledge from website documents using data mining methods and techniques (Etzioni, 1996). It is an integrated field involving a few research areas, such as informatics, statistics, data mining, and computational linguistics. Although data mining research started more than two decades ago, it became more significant because of the dramatic growth of the availability of information resources on the web (Berardi et al., 2015; Kosala & Blockeel, 2000). The state-of-the-art decomposes web mining into three categories: web content mining, web structure mining, and web usage mining (Anami, Wadawadagi & Pagi, 2014; Kosala & Blockeel, 2000).

Web content mining focuses on the contents of a website itself. Websites contain several data types; text, hypertext, image, video, and audio, which together constitute a topic or more inside the webpage. Literature has utilized topic modeling on web content data for various applications, such as clustering (Alharbi, Hijji & Aljaedi, 2021; Zhao et al., 2018), classifying (Gu et al., 2016), detecting and filtering (Asdaghi, Soleimani & Zahedi, 2020; Liu & Forss, 2015b), and ranking web pages (Lee & Cho, 2021).

Several studies have proven the benefit of topic modeling in classifying and clustering web pages and websites (Gu et al., 2016). The training of a topic model on a large corpus of web pages is a standard method for web classification using topic modelling. This entails treating each web page as a bag of words in which each word is handled as an individual vector. A recent study has proven the impact of topic modeling to cluster web pages using LSA and PLSA (Alghamdi & Selamat, 2015). Other studies have used the LDA topic model to enhance the classification of harmful and inappropriate web pages using their extracted content (Liu & Forss, 2015a; Liu & Forss, 2015b). Similar studies detect spam webpages using the LDA topic models (Asdaghi, Soleimani & Zahedi, 2020; Wan et al., 2015). These studies only utilize the LDA topic model in their applications. Chen and Zhou have modified the LDA model to include user-related tags in order to enhance web clustering (Chen & Zhou, 2014). The authors (Sayadi, Bui & Bui, 2015) combined the LDA topic modeling with the random forest classifier to perform multilayer soft web classification. Other studies address clustering non-English webpages using the LDA (Alharbi, Hijji & Aljaedi, 2021). A recent study has utilized the LDA and word2vec to classify webpages based on their ranking (Lee & Cho, 2021).

Topic modeling is also utilized to discover trending topics in a specific subject area or speciality. Several recent studies have used the LDA topic model to discover the popularity of topics in a specific field, such as the IoT and Industry 4.0 (Shah, Naqvi & Jeong, 2021), terms of service (Sundareswara et al., 2021), and marketing travelling blogs (Shan-shan, Guo-ming & Xi-tong, 2013). Although topic modeling does not directly detect fake news, it has been utilized to identify the content of news articles and, therefore, detect potential sources of fake news (Kim, Park & Mariani, 2023; Xu et al., 2019).

Topic modeling also identifies the most common topics that are being discussed in Question and Answer (Q&A), rating, and review websites. Shah, Naqvi & Jeong (2021) used dynamic topic modeling to identify dominant topics related to COVID-19 health issues. A similar approach was applied using the LDA model to detect and summarize topics of gaming development (Kamienski & Bezemer, 2021), development of security vulnerability (Le et al., 2021), and startup entrepreneurship (Nurhas et al., 2021).

A common drawback of the abovementioned studies is that they have only utilized topic modeling based on textual documents that have been extracted from web pages. However, they have not considered the different structures of web content data. Neglecting the unique structure of web content leads to missing the otherwise topics and, therefore, low topic quality, as the first experiment demonstrates in Section 5.1.

A few recent studies, however, have addressed the unique structure of web content when applying topic modeling. Yang et al., (2020) proposed a distribution topic model, referred to as Named Entity Topic Model (NETM), to extract web content popularity growth factors. Although the NETM has achieved higher accuracy compared to the LDA model, it has not addressed the HTML structure of the webpage content. Another interesting and recent study has been proposed by Zhao et al. (2021), where the authors proposed a topic-graph probabilistic personalization model for web search based on the LDA model. The model includes the relevancy of the webpage based on the previous probabilities of retrieving a relevant or non-relevant webpage. The model has proven its effectiveness compared to the benchmark models; however, it neglects the HTML structure of the webpage. Neglecting the unique structure, ss the first experiment in Section 5.1. indicates, causes to missing produce a low topic quality.

## HTM Topic Model

A web page represents web contents in a hypertext document provided by a website, usually comprising several web pages. HTML tags represent webpages, which constitute their hyperlinked structures and their textual contents. These tags, such as <title>, <metadata>, <a>, <b>, <ul>, <li>, <hr>, and <img> normally contain very short textual contents. Unlike conventional text documents, combining these textual contents from these tags results in a sparse and incoherent document. The sparseness and incoherence create challenges and cause the traditional text mining and topic model methods to be ineffective for web content mining (Figueiredo et al., 2013).

The HTM topic model is based on the LDA and aims to enhance the performance of topic modeling for web content-based data. The HTM topic model considers the structure of the textual contents within the HTML tags to extract topics from a web page. HTML tags are usually used to add textual content to the webpage. The HTML tag element consists of a start tag, end tag, attribute name, attribute value, and textual content, as Fig. S2 illustrates. All HTML tags that contain visible textual content are considered by the HTM topic model. in general, the HTM model extracts only the visible textual content of each HTML tag element of the webpage and uses it as a document within a webpage and each webpage in a website as a document. The extracted topics of these tags’ textual content can describe the webpage efficiently and, therefore, create practical web topic modeling. Besides that, HTML attributes which provide additional information with visible textual content are also considered. These attributes are alt, title, label, value, placeholder, and data-*attributes. The following subsection describes the generative process of the HTM model.

### Problem formalization and notations

This section describes the problem formally and addresses the used notations in the following subsections. The definition of the problem is as follows:

Consider a collection of webpages

(1) \begin{eqnarray*}Dataset(D)=\{ W{P}_{0},W{P}_{1},\ldots ,W{P}_{p-1}\} \end{eqnarray*}

where *WP*_*i*_ is the i-th webpage of a dataset collection D, and p is the number of web pages in the collection. Each of these web pages is composed of HTML tags

(2) \begin{eqnarray*}Webpage~(WP)=\{ T{G}_{0},T{G}_{1},\ldots ,T{G}_{t-1}\} \end{eqnarray*}

where *TG*_*i*_ is the i-th HTML tag of a webpage WP and t is the number of HTML tags in the webpage.

An HTML tag topic in a given webpage is the distribution of all words relating to this webpage and can be represented as,

(3) \begin{eqnarray*}{\theta }_{tg}={ \left\{ {{\theta }_{tg}}_{i} \right\} }_{i\in {1}_{tg}}\sim Dir \left( \cdot {|}{\alpha }_{wp} \right) .\end{eqnarray*}

Taking a sports news webpage as an example, which includes many HTML tags, each may include different sub-topics. However, some tags in the webpage may include some other recent news and the side of the webpage for users to read. This news can be related to sports as well as other topics such as political news, health news, and many others. In this case, taking the webpage as one piece could give low topic coherence and, therefore, generate low topic quality. The webpage topic modeling problem aims at finding topics that occur on a webpage and ensures that the generated topics are semantically coherent. The final goal of this article is to learn coherence topics in web content data for a given webpage.

Before introducing the generative process and the mathematical explanation of the model, Table 1 tabulates the used notations.

**Table 1:** Description of the used symbols in the HTM topic model.

Symbol	Description
α	Per-document topic distributions
β	Per-topic word distribution
θ_1_	Topic distribution for *TG*
θ_2_	Topic distribution for *WP*
*ψ*	Word distribution for *T*
Z	Topics of the n-th word in *TG*
W	Specific word
V	Set of words in the vocabulary
WP	Webpage
TG	HTML Tag
N	is the number of words in a given document

### The generative process

As the LDA topic model, the HTM model is based on a generative statistical model, and it uses latent factors to capture the semantic similarities of words and documents. The generative process of the HTM model is as follows. Firstly, we need to specify the optimal number of topics represented by (K). Then randomly choose a distribution over topics (a multinomial of length K). A specific webpage (WP) is modelled as a sequence of words W = (W_1_, …, W_ℓ_) of length ℓ ∼Poisson(*ξ*), where *ξ* is pre-specified. For this webpage WP, a K-dimensional probability vector *θ* with non-negative coordinates summed to one is used to model the topic mixture. Three probability distributions are assumed to be multinomial distributions: p(z—wp), p(z—tg), and p(w—z). Therefore, the topic distributions in all web pages share the common Dirichlet prior *α*, and the word distributions of topics share the common Dirichlet prior *β*. Given *α* and *β* as the parameters for webpage WP, parameter *θ*_wp_ of a multinomial distribution over K topics is constructed from Dirichlet distribution Dir(*θ*_wp_— *α*). The alpha is initiated parameter for the webpage (theta_{wp}), and for the simplicity of the distribution, the same parameter is then used for the tags within a specific webpage. Notice that the vector size of alpha will differ within each webpage based on its tags. Therefore, parameter *θ*_tg_ of a multinomial distribution over K topics is constructed from Dirichlet distribution Dir(*θ*_tg_— *α*). For topic t, parameter *φ*_t_ of a multinomial distribution over the set of words in the vocabulary (V) is derived from Dirichlet distribution Dir(*φ*_t_— *β*). The Dirichlet distribution is a convenient choice as a prior and can simplify the statistical inference in the HTM model. The likelihood is multiplied through all the web pages and maximized with the technique of variational inference for the estimation of *α* and *β*.

A summary of the generative process for a set of web pages is as follows:

*For each topic* $t\in \left\{ 1,\ldots ,T \right\} $

*Generate* ${\phi }_{t}={ \left\{ {\phi }_{tw} \right\} }_{w=1}^{V}\sim Dir \left( \cdot {|}\beta \right) $

*For each web page* $WP\in \left\{ 1,\ldots ,N \right\} $

*Generate* ${\theta }_{wp}={ \left\{ {{\theta }_{wp}}_{i} \right\} }_{i\in {1}_{wp}}\sim Dir \left( \cdot {|}{\alpha }_{wp} \right) $

*For each HTML tag tg in the webpage WP*

*Generate* ${\theta }_{tg}={ \left\{ {{\theta }_{tg}}_{i} \right\} }_{i\in {1}_{tg}}\sim Dir \left( \cdot {|}{\alpha }_{wp} \right) $

*For each word w in the HTML tag tg*

*Generate* ${z}_{tgn}\in { \left\{ {{\theta }_{tg}}_{i} \right\} }_{i=1}\sim Multinominal \left( \cdot {|}{\theta }_{tg} \right) $

*Generate* ${w}_{tgn}\in \left\{ 1,\ldots ,V \right\} \sim Multinominal \left( \cdot {|}{\phi }_{{z}_{tgn}} \right) $

The following snapshots elaborate more on how a webpage will transform from metadata to preprocessed data ready to be inserted into the HTM topic model. Figure S3 shows a snapshot from webpage metadata. Notice that the metadata contains various HTML tags, such as <div>, <span>, <svg>, <p>, and <h4>. The HTM assumes that the webpage is a distribution of tags; therefore, each tag will be preprocessed separately. Notice that only visible text tags will be used, and their textual content will be extracted, as explained in Fig. S3. Figure S4 shows a snapshot of the preprocessed textual data, where each tag is represented separately as a list. The following section represents these steps mathematically and elaborates on the role of the tags of a webpage using a plate notation of the HTM topic model.

### Mathematical model

**Figure 1:**
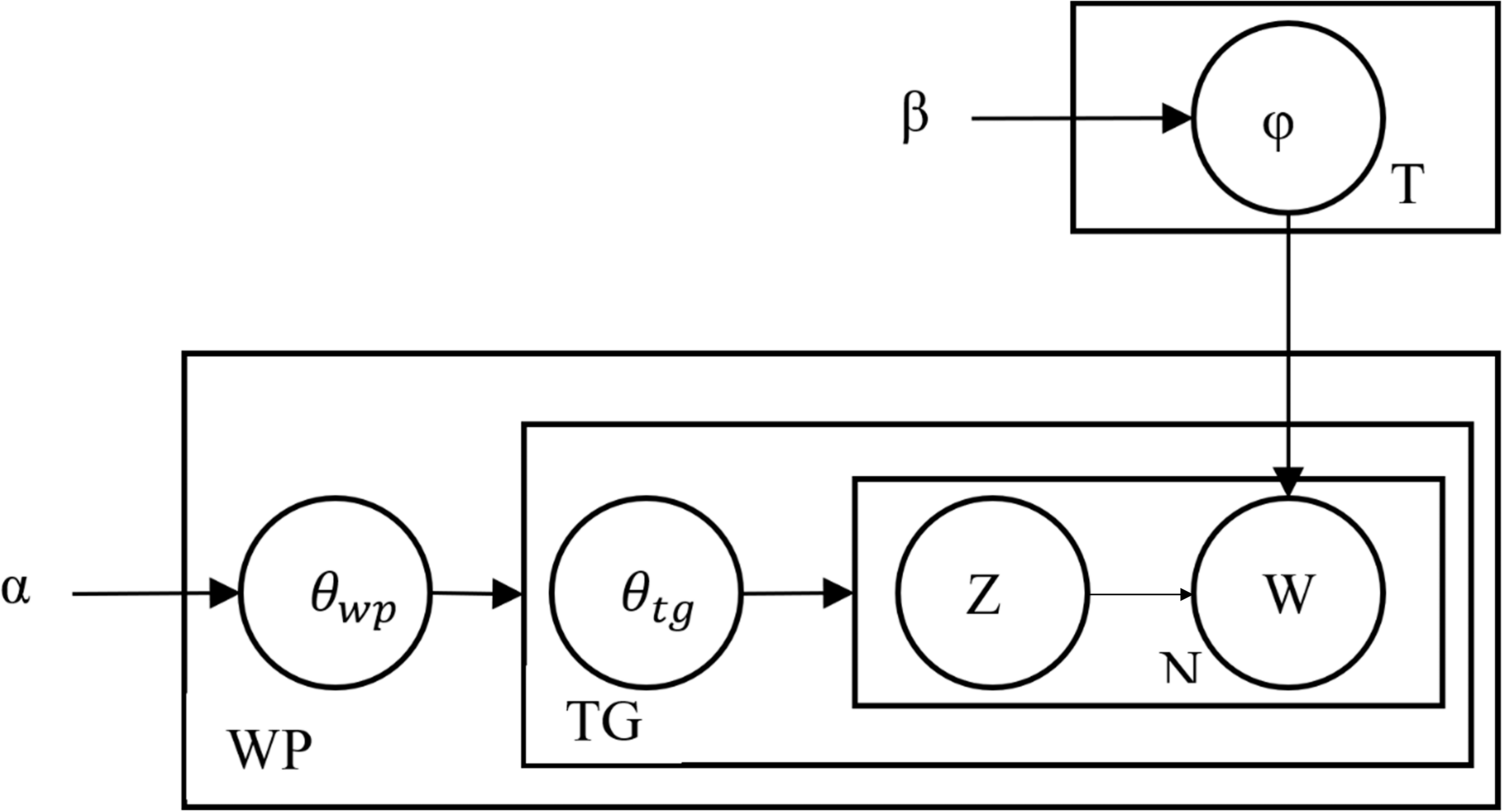
Plate notation of the HTM topic model.

The HTM topic model, like the LDA model, is based on the probability distribution model. Figure 1 shows the graphical model of the HTM topic model, referred to as the plate notation graph.

The model infers the distribution of the hidden variables by using the joint probability distribution. This inference aims to approximate the posterior $\rho \left( \beta ,\theta ,\mathrm{Z} \left\vert W \right. \right) $ with the distribution $q \left( \beta ,\theta ,\mathrm{Z} \right) $ using the variance inference, simplifying the model analysis. Figure S5 illustrates the inner plate representing the probability distribution of words per topic to simplify the model.

In this sub-graph, *β* acts as a global variable, while $ \left. \mathrm{Z} \right\vert W$ acts as a local variable for each word in the corpus. This part is inherited as it is from the LDA model. The mathematical definition of this plate is as follows:

(4) \begin{eqnarray*}p \left( \beta ,{Z}_{1:n},{W}_{1:n} \right) =p \left( \beta \right) \prod _{i=1}^{n}p({Z}_{i}{|}\beta )p{W}_{i}{|}{Z}_{i},\beta .\end{eqnarray*}

This sub-graph is associated with the per-HTML tag topic proportion variable and the word distribution for each topic. Figure S6 illustrates this association of the model.

The parameter *α* of the Dirichlet distribution models the topic distribution variable *θ*_*tg*_ per HTML tag, while the parameter *φ* of the multinomial distribution models each specific associated topic Z_*i*_. This association is defined as:

(5) \begin{eqnarray*}q \left( \beta ,{\theta }_{tg},Z \right) =\prod _{k=1}^{K}q({\beta }_{k}{|}{\varphi }_{k})\prod _{tg=1}^{TG}q({\theta }_{tg}{|}{\alpha }_{tg})\prod _{n=1}^{N}q({Z}_{tg,n}{|}{\varphi }_{tg,n}).\end{eqnarray*}

Once the HTM model processes HTML tags, the model then applies a similar step on all the given web pages. The following equation describes the process as follows:

(6) \begin{eqnarray*}q \left( \beta ,{\theta }_{wp},{\theta }_{tg},Z \right) =\prod _{t=1}^{T}q \left( {\beta }_{t}{|}{\varphi }_{t} \right) \prod _{wp=1}^{WP}q({\theta }_{wp}{|}{\alpha }_{wp})\prod _{tg=1}^{TG}q \left( {\theta }_{wp,tg}{|}{\alpha }_{wp,tg} \right) \prod _{n=1}^{N}q({Z}_{tg,n}{|}{\varphi }_{tg,n}).\end{eqnarray*}

Once the HTM model processes all the web pages, the model then updates the parameters of the topics $ \left( \varphi ~and~\alpha \right) $. The model updates these parameters after each iteration. In each iteration, as the *α*_*tw*_ value increases, the chance of selecting the word W from the HTML tag *TG* in topic *T* also increases.

## Experiments Setup

We conducted two series of experiments to evaluate the topic coherence of the HTM in comparison to the benchmark topic models. The methodology of each experiment consists of an objective, datasets, data preprocessing, model implementation, evaluation measures, and model evaluation and comparison with benchmark models.

The first experiment aims to demonstrate the drawbacks of the existing topic models when applied to web content data. This study uses two data sources to exhibit these drawbacks: conventional data and web content data, described in detail in the subsequent subsections. Both datasets were preprocessed using four steps commonly used in text data for topic modeling, explained in the subsequent subsections. We used the Python programming language with the help of the Anaconda distribution platform, Gensim library (Rehurek & Petr, 2011), and Tomotopy package (bab2min & Jonathan, 2021) to implement each of the benchmark models and run it to model topics in each data source. During the implementation of the models, parameters such as alpha, beta, random state, chunk size, and passes are set to default values. We then used topic coherence evaluation metrics to illustrate the performance of each topic model on each data source, explained in the following subsections. We then compared the results of the benchmark topic models on each data source for each evaluation measure. We selected the best-performing model to compare with our proposed model in the second experiment.

The second experiment aims to evaluate the effectiveness of the HTM model in discovering coherent patterns of web content data. In this experiment, we used the web content dataset only, and the preprocessing steps were similar to the previous experiment, described in detail in the subsequent subsections. We use the Python programming language with the help of the Gensim library to develop the HTM model. We executed the HTM model on web content data and evaluated the results using the same evaluation measures of the previous experiment. The results of the HTM model and the chosen model from the previous experiment are then compared.

Further details regarding the adopted evaluation measures and data sources used in both experiments are provided in the subsequent subsections.

### Performance evaluation

Literature in the field of topic modeling uses perplexity measure, topic coherence measure, or both measurements. Several recent studies have argued that perplexity is less correlated to human interpretability and understandability (Li et al., 2016; Röder, Both & Hinneburg, 2015; Zuo et al., 2016) and does not address the goal of exploratory research of topic modeling. Thus perplexity is no longer a general way of evaluating topic models (Zuo et al., 2016). On the contrary, topic coherence metrics have proven to correlate with human judgments and interpretability (Kim, Park & Lee, 2020; Law et al., 2017; Röder, Both & Hinneburg, 2015; Syed & Spruit, 2017). Taken these considerations together, this study evaluates the benchmark topic models and the HTM topic models using topic coherence metrics.

Topic coherence can calculate and measure the consistency and quality of each individual topic with reference to the semantic similarity between the words in the topic or how many words of each individual topic occur within the same set of documents (Li et al., 2016; Mimno et al., 2011). The authors (Mimno et al., 2011) introduced the topic coherence metric, which strongly correlates with human judgements in evaluating topic quality (Chen & Zhou, 2014). Topic coherence indicates the quality of the model and how accurate the terms are. The higher the topic coherence score, the more efficient the model. Topic coherence metrics often rely on the method of sliding window. Sliding refers to the step-by-step movement of a window with a defined size over a text corpus. It permits the metric to recognize local patterns in the text and calculate coherence scores for each window. The window concept is used to determine the scope of the coherence measure for a specific topic. It refers to the subset of documents or terms utilized to calculate the topic’s coherence score. The window size determines the number of words or terms in each window. For instance, a window size of 10 indicates that each window includes 10 consecutive text words. The commonly used coherence calculations in the literature are C_UMass_ (Mimno et al., 2011), C_UCI_ (Newman et al., 2010b), C_NPMI_ (Bouma, 2009b), and C_V_ (Lau, Newman & Baldwin, 2014; Syed & Spruit, 2017). The following experiments are evaluated by using these metrics.

### Dataset

This section describes the datasets used in the experiment. This study investigates differences in the performance of topic models on conventional document/article data and webpage content data. We use two datasets, one for each type of data, to achieve this aim. The following subsections (1 and 2) introduce each dataset and describe its features and sources. Both datasets are then preprocessed for evaluation, as subsection 3 illustrates.

#### Conventional document-based dataset

Recent studies have used articles from well-known sources such as Wikipedia, Reuters, BBC, and the New York Times to evaluate topic models (Fu et al., 2021; Ma et al., 2020; Röder, Both & Hinneburg, 2015; Syed & Spruit, 2017; Wang, He & Yang, 2020). Wikipedia provides a copy of the whole wikitext source and embedded metadata in a single XML file (https://dumps.wikimedia.org/enwiki/latest/). We chose the latest English page articles at the time of obtaining the dataset (22-Feb-2023). The size of the file was 20,580,560,896 bytes, containing more than 7 million articles. These articles include topics from various Wikipedia categories such as art and culture, geography and places, health and fitness, history and events, mathematics and abstractions, natural sciences and nature, people and self, philosophy and thinking, religion, social sciences and society, and technology and applied sciences category. For the first experiment, the study randomly chooses 50,000 articles to represent the Conventional Document-based (CD-based) dataset. The CD dataset contains 55,775,941 total words with *V* = 584,132 unique words. The average of words per document was about 1,115.

#### Web content-based dataset

This study uses web content data to evaluate the performance of the benchmark topic models and the HTM model. We use an available dataset containing 2 million web pages collected from about 7,000 websites (Altarturi & Anuar, 2022). These websites were collected from DOMZ and Alexa, which both contain their web categories. Examples of such categories that this dataset contains are arts, business, computers, games, health, news, science, society, sports, and kids & teens. The variety of these categories reflects the variety of topics on these web pages. We randomly choose 125,000 web pages to represent the Web Content-based (WC-based) dataset. The WC dataset contains 55,753,919 total words with *V* = 400,230 unique words. The average of words per document was about 446.

Webpages, however, contain HTML content, which requires an additional step to extract the textual content. Extracting webpage content requires a web scrap agent to crawl and parse the URL of the webpage. Web crawling aims to index the entire web pages contained in a specific website by systematically browsing the World Wide Web. The parsing HTML code extracts relevant web page contents such as paragraphs, images, bold texts, web page titles, refs, and metadata.

Although there are several ways to scrape a website, *Python* offers the most flexible and powerful way to do it. A few Python libraries support web crawling and scraping, such as *BeautifulSoup*, *LXML*,* MechanicalSoup*,* Requests*,* Scrapy*, and *Urllib*. In fact, building an automatic and systematic website crawler and scrapper requires using a combination of these libraries. This study uses a Python library called CrawlerScraper to create the dataset from the collected websites. The CrawlerScraper is an open-source library for the solution of efficient and easy web crawling and data scraping (Altarturi, 2022).

#### Data preprocessing

Data preprocessing is an essential step in machine learning and data mining in general. For topic modeling, this task assures the quality and clarity of the resulting topics. In order to transfer the selected documents into meaningful and formatted data, this task consists of four steps which are explained as follows:

1. Text tokenization. Tokenization is the action of splitting the text into sentences and the sentences into words. Words are then lowercase, and punctuation marks are removed.
2. Stopwords removal. Stopwords are English words that do not add much meaning to a sentence. They can safely be ignored without sacrificing the meaning of the sentence. This step also includes removing all special characters and words such as email signs, newlines, and quotes.
3. Bigram constructing. A bigram, a particular form of n-gram with two adjacent elements, is a probabilistic model that aims to predict the different meanings of words when they are in a sentence. This step is essential because sometimes word groups are more beneficial in explaining the meaning than single words.
4. Word lemmatization. Lemmatization, a special case of normalization, aims to reduce the inflectional forms and sometimes derivationally the related forms of a word to a common base form. This step maintains the part-of-speech (POS) tagging.

## Results and Discussion

This section shows the results of both experiment series of this study. As aforementioned, the first experiment series demonstrates only the benchmark models’ performance limitation when applied only to web content data. The following subsections firstly address this experiment’s results based on this study’s performance evaluation metrics. The second experiment evaluates the enhancements made by the HTM model in discovering coherent patterns of web content data. The following subsection then illustrates this experiment’s performance evaluation metrics’ results.

### Benchmark models evaluation

We used the Python programming language with the Anaconda distribution platform’s help to evaluate CTM, DMR, HLDA, LDA, PTM, and sLDA based on both CD-based and WC-based datasets. We used the topic coherence metrics to evaluate a topic model’s quality and performance. This section evaluates the topic models using C_UMass_, C_UCI_, C_NPMI_, and C_V_ metrics. Using these metrics, the performance of the models for each dataset and given the number of topics K ∈{1…,100} will then be compared in the following subsections. Section 5.1.4 then discusses the results of compassion.

#### First experiment’s results and discussion

**Figure 2:**
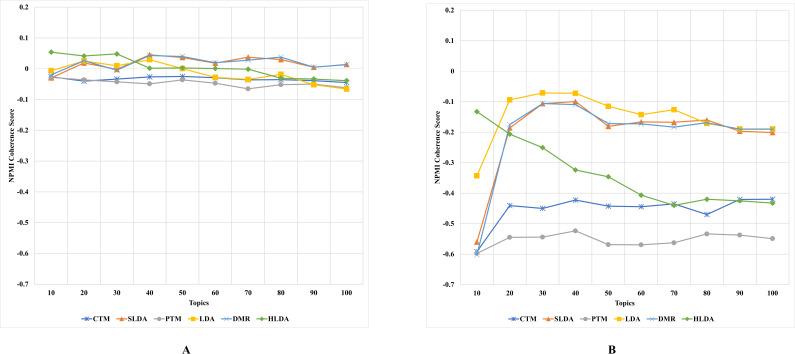
C_NPMI_ topic coherence of the topic models on both CD-based (A) and WC-based (B) datasets.

**Figure 3:**
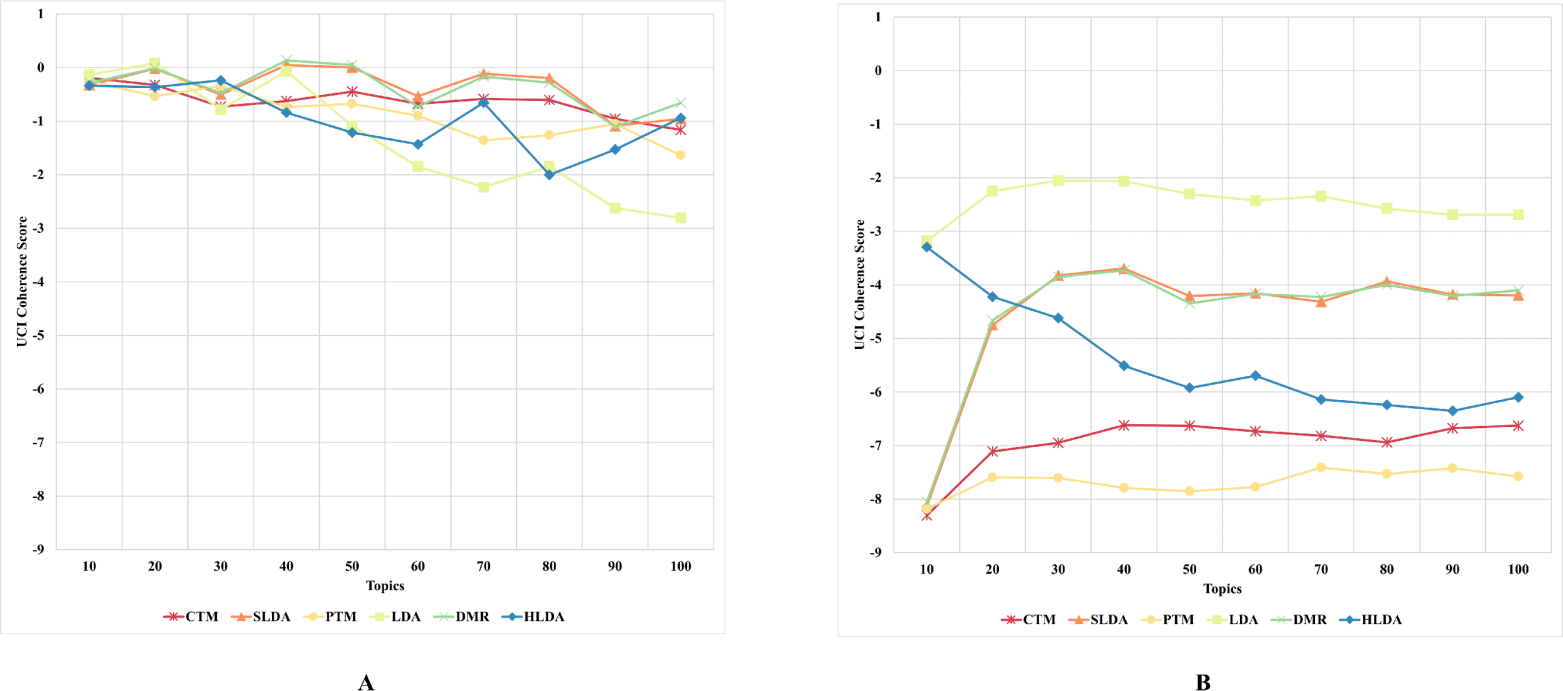
C_UCI_ topic coherence of the topic models on both CD-based (A) and WC-based (B) datasets.

**Figure 4:**
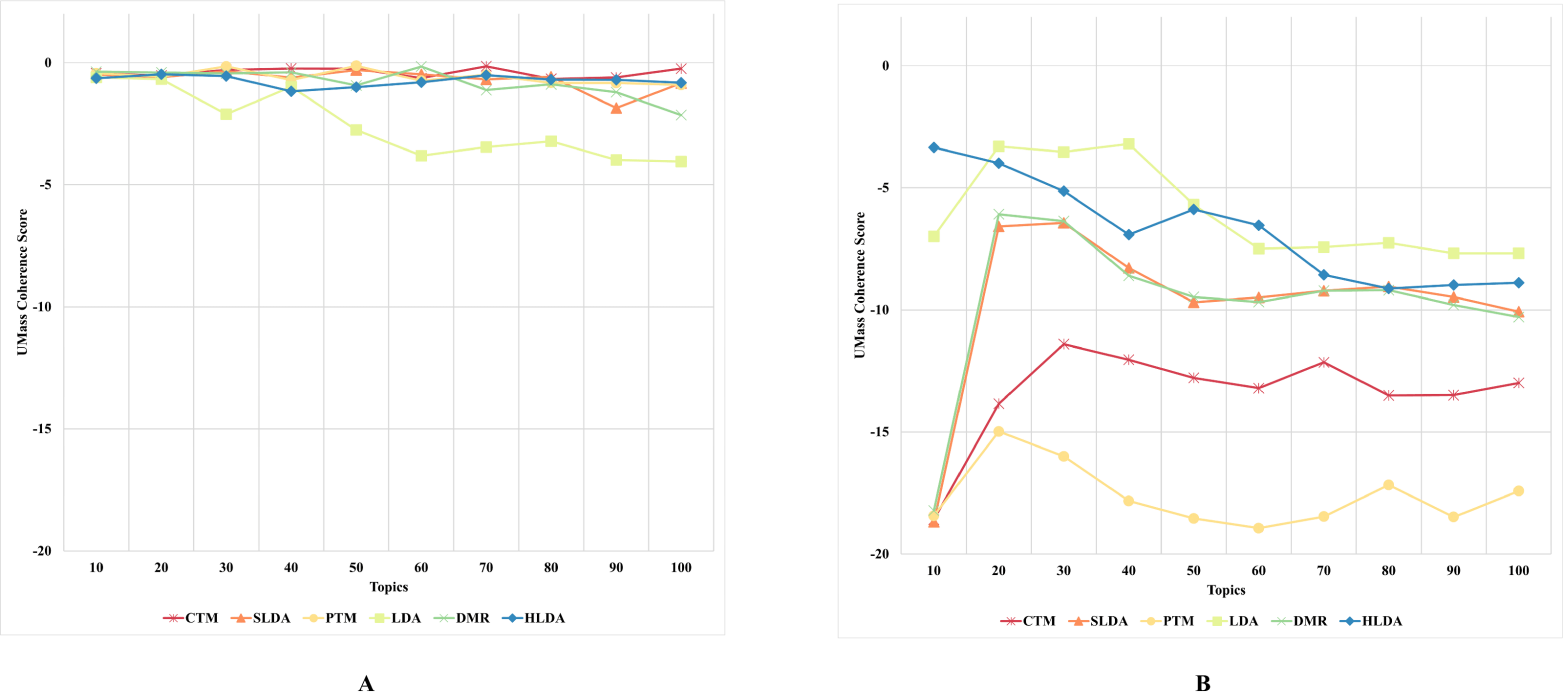
C_UMass_ topic coherence of the topic models on both CD-based (A) and WC-based (B) datasets.

**Figure 5:**
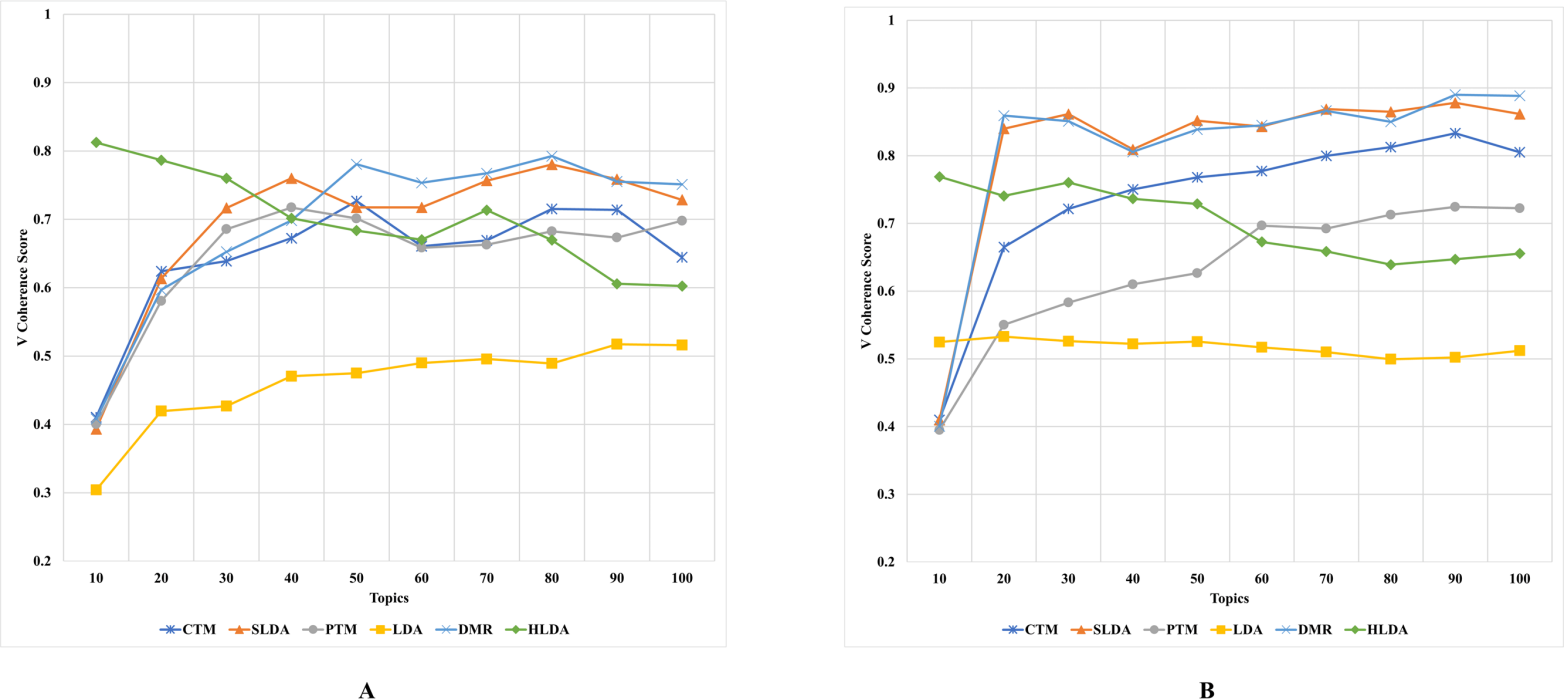
C_V_ topic coherence of the topic models on both CD-based (A) and WC-based (B) datasets.

The results, as depicted in Figs. 2–5 (where A is the performance on CD-based dataset and B is the performance on WC-based dataset), showed that the overall performance of these topic models on conventional document data outweighs their performance on documents with web content-based data across the number of topics. These results indicate that the benchmark models failed to capture the coherence of topics on webpage content data. The difference in performance was significant for some topic models, such as the PTM and CTM, and it was inconsiderable for other models, such as the LDA. This result answers the first research question of this study. The C_UCI_ and C_NPMI_ topic coherence appear to prefer fewer topics on conventional documents data. In contrast, the C_V_ topic coherence favours a higher number of topics for all models except the HLDA model. The results suggest that the optimal number of topics may vary depending on the evaluation metric used and the type of dataset being analyzed.

An interesting observation was made regarding the LDA model’s performance on both datasets. Although it performed the worst for the CD-based dataset, it performed the best for WC-based datasets, achieving the highest topic coherence score in comparison to other models. This indicates that the LDA model is the most stable model among the benchmark topic models based on the C_UMass_, C_UCI_, and C_NPMI_ metrics. This result answers the second research question of this study.

Although the CTM model consistently achieved the highest topic coherence score given different numbers of topics for CD-based datasets, it witnessed the largest failure among the benchmark topic models when evaluated using the CUCI metric on web content data. Similarly, when evaluating the C_NPMI_ metric, the sLDA and DMR models achieved the highest topic coherence scores given different numbers of topics for CD-based datasets, whilst the PTM model scored the lowest. It is worth mentioning that the sLDA scores are similar to the DMR scores given all metrics and in both datasets, which is likely due to their methodological similarities (Mimno & McCallum, 2008). Although the authors (Mimno & McCallum, 2008) emphasize the difference between their proposed DMR model and the sLDA model, both models perform comparably similarly in our study.

Another interesting observation was that the DMR and sLDA models performed the best on both CD-based and WC-based datasets based on the C_V_ metric. However, it is worth noting that although all benchmark models perform better on WC-based datasets than CD-based datasets, the increase in topic coherence score is modest, indicating only a slight enhancement.

This finding is somewhat surprising since one might expect models to perform better on web content data, given this data’s relatively free-form and less-structured nature. However, the results indicate that neglecting the web content data structure results in low-coherence topics, thus generating low-quality and semantic topics from web content data.

### HTM evaluation

This study presents an analysis of the topic modeling of web content data using the LDA and HTM models. The goal of the HTM topic model is to improve the performance of the LDA model, which is a widely used topic modeling algorithm. The study evaluates both models using four coherence metrics: C_UMass_, C_UCI_, C_NPMI_, and C_V_, and compares their performance for different numbers of topics (K ∈ {1…,100}). We used the Python programming language with the help of the Gensim library to perform data preprocessing and implement the LDA model and the HTM topic model.

#### Second experiment’s results and discussion

**Figure 6:**
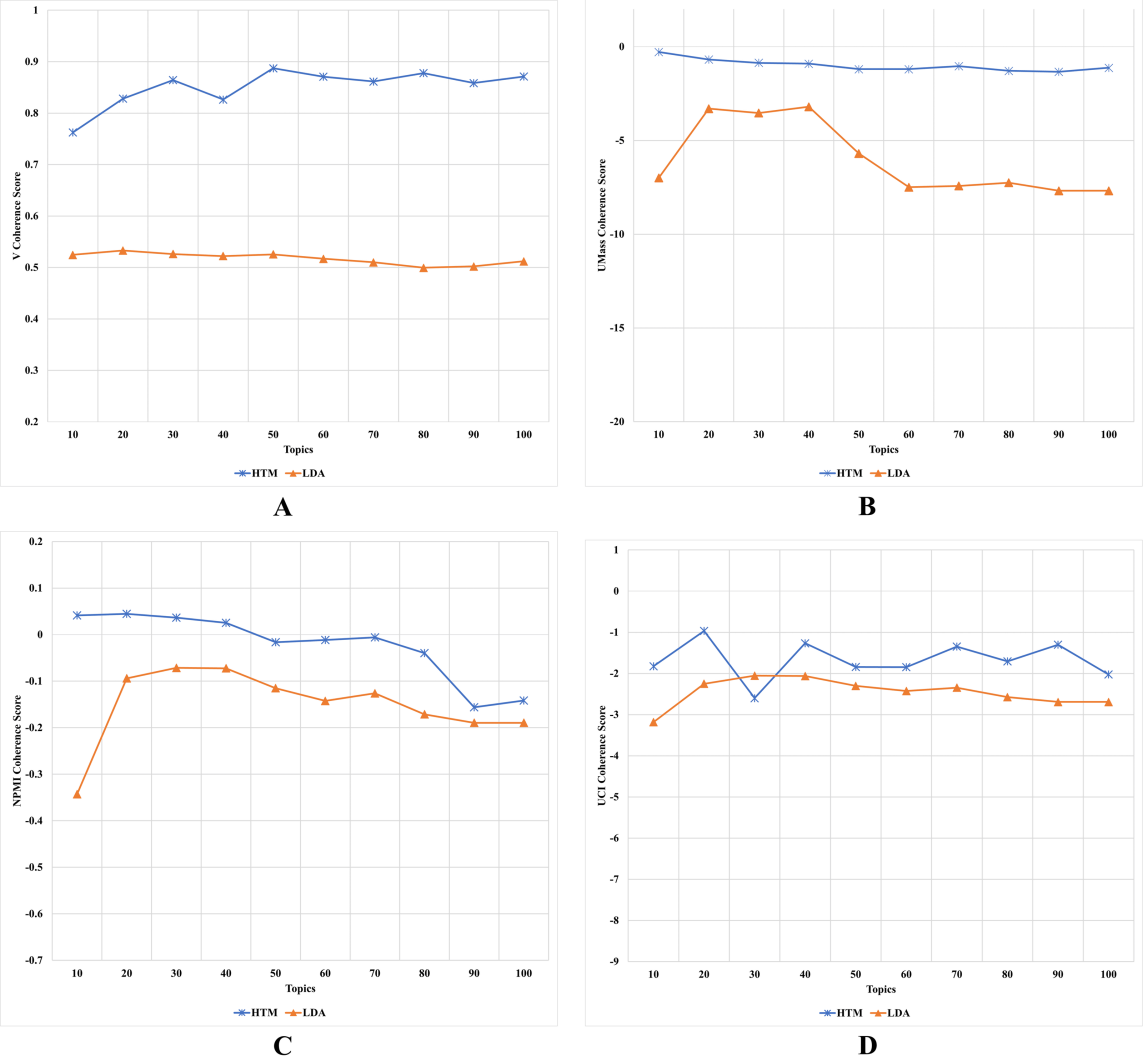
(A–D) Each topic coherence score of the HTM and LDA topic models.

The results, as depicted in Fig. 6 (where A is based on C_V_ metric, B is based on C_UMass_ metric, C is based on C_NPMI_ metric, and D is based on C_UCI_ metric), indicate that the HTM model outperforms the LDA model in terms of overall performance. The C_UMass_ coherence score shows that the HTM model has a significantly better performance than the LDA model, with a steady value over the number of topics, while the LDA model’s value decreases as the number of topics increases. The improvement of the HTM model was slightly more than 89% of the LDA using the C_UMass_ metric. This is due to the UMass metric, which considers preceding and succeeding terms in the list and allows the HTM model to relate topics within each HTML tag and among tags on each webpage. Similarly, the C_V_ coherence score shows that the HTM model outperforms the LDA model for any number of topics, with a C_V_ value of ≥ 0.9 when the number of topics exceeded 4. The improvement of the HTM model was slightly more than 36% of the LDA using the C_V_ metric. These results indicate that the HTM model learns better web content topics than the LDA model, which may enhance human interpretability, as some previous studies argued (Mimno et al., 2011; Newman et al., 2010a; Röder, Both & Hinneburg, 2015). These results indicate that considering HTML tags when applying topic modeling on web content data increases the quality of generated topics, which answers the third research question of this study.

The C_UCI_ and C_NPMI_ coherence scores show that the HTM model performs slightly better than the LDA model when the number of topics is high. However, when the number of topics is low, the HTM model significantly outperforms the LDA model. Another improvement of the HTM model was recorded using the C_NPMI_ metric with about more than 26%. This is due to the fact that the HTM model considers the indirect coherence between related terms of each tag, which enhances its performance in learning coherence topics.

The results show that the enhancement made by the HTM model was vast based on C_UMass_ and CV metrics yet slight based on CUCI and C_NPMI_ metrics. These phenomena suggest that the HTM model made a significant enhancement of the LDA model in generating topics that are semantically related and coherent in terms of the overall corpus. However, the HTM model was similar to the LDA model in terms of generating a strong association within each topic. This result is due to several reasons related to the nature of each coherence metric.

C_UMass_ coherence measures the degree of semantic coherence between the words in a topic by comparing the observed co-occurrence of words within the topic to their expected co-occurrence in a reference corpus. C_V_ coherence measures the degree of coherence based on the exclusivity of the top words in a topic. Both UMass and V coherence metrics often indicate how well the topic model captures global patterns in the corpus (Lau & Baldwin, 2016).

C_UCI_ coherence and C_NPMI_ coherence, on the other hand, measure the degree of association between word pairs within a topic. C_UCI_ coherence calculates the pointwise mutual information (PMI) between the words in the topic, while C_NPMI_ normalizes PMI by dividing it by the negative logarithm of the probability of the word pair occurring together by chance. Both C_UCI_ and C_NPMI_ coherence metrics are based on the PMI, which often indicates how well the topic model captures local patterns in the corpus (Bouma, 2009a).

In conclusion, this study demonstrates that the HTM model outperforms the LDA model in the topic modeling of web content data and provides evidence of the benefits of the HTM model in learning coherent topics. These findings are useful for researchers in the field of web content analysis and topic modeling. These finding also answers the fourth research question of this study. A summary of these coherence metrics results is represented by Fig. S7.

## Conclusion and Future Work

Topic detection in the contents of the webpage is a crucial and remarkable research problem in natural language processing. This study compared the CTM, DMR, HLDA, LDA, PTM, and sLDA topic models on both the CD-based dataset and the WC-based dataset. The comparison showed the drawback of the performance of these topic models on the web content-based data. Among these models, the LDA outperformed in many ways by appearing among the top two models per C_UMass_, C_UCI_, and C_NPMI_ metrics on the web content-based data. This comparison indicated the need for a topic model that addresses the speciality of web content-based data. The study introduced the HTM topic model, a web content-based topic modeling method, which enhances the LDA model. The experimental results show that the HTM model outperforms the LDA model over the web content-based data regarding C_UMass_, C_NPMI_, and C_V_ coherences.

This study proved that considering the HTML tags in topic modeling generates high-coherence topics, thus, higher topic quality for web content data. This opens the door and creates a future research prospect for developing the HTM topic model concept based on recent topic models and word embedding models. In future work, we suggest evaluating the performance behaviour of the HTM topic model, benchmark models, and embedding models based on different types of web pages. It is also suggested to include the comparison with non-probabilistic topic modeling approaches such as BERTopic model. Evaluating the interpretability of the HTM model and other models when applied on web content data is also a useful aspect in future work. We also suggest including several features that the current HTM model does not address, such as handling XHTML format documents and investigating the weight of different HTML tags (<title>, <metadata>, <a>, <alt>, and <img>) in topic modeling. It would also be very interesting to utilize the HTM topic model in web mining methods and techniques such as classification, clustering, and association rules. Future work can also address additional evaluation of the HTM topic model on the different webpages categories, such as social media, news and magazines, blogs, directories, landing, portfolios, homepages, and e-commerce webpages. Finally, it might be beneficial to include the web structure mining concept in the HTM topic model. Considering the parent, siblings, and children pages may enhance the quality of the generated topics of a webpage.

##  Supplemental Information

10.7717/peerj-cs.1459/supp-1 Supplemental Information 1 Characteristics and limitations of the benchmark topic models

10.7717/peerj-cs.1459/supp-2 Supplemental Information 2 Topic models categorized based on their underlying statistical models

10.7717/peerj-cs.1459/supp-3 Supplemental Information 3 HTML tag element

10.7717/peerj-cs.1459/supp-4 Supplemental Information 4 Raw HTML content of a webpage

10.7717/peerj-cs.1459/supp-5 Supplemental Information 5 Pre-processed HTML content of a webpage

10.7717/peerj-cs.1459/supp-6 Supplemental Information 6 Inner plate notation of the HTM topic model

10.7717/peerj-cs.1459/supp-7 Supplemental Information 7 Outer plate notation of the HTM topic model

10.7717/peerj-cs.1459/supp-8 Supplemental Information 8 Overall topic coherence scores of the HTM and LDA topic models

## Additional Information and Declarations

### Data Availability

The following information was supplied regarding data availability: The data is available at Mendeley: Altarturi, Hamza (2022), “Ground truth dataset: objectionable web contents”, Mendeley Data, V2, doi: 10.17632/f239556fkr.2
The code is available at GitHub and Zenodo: https://github.com/hamzatartori/CrawlerScraper

https://github.com/hamzatartori/HTML-Topic-Model. Hamza Altarturi. (2023). CrawlerScraper. Zenodo. https://doi.org/10.5281/zenodo.8022087
Hamza Altarturi. (2023). HTML Topic Model (HTM) (V.1). Zenodo. https://doi.org/10.5281/zenodo.7992951
