# Supplementary material for: Web content topic modeling using LDA and HTML tags
Source: PeerJ Comput Sci. 2023 Jul 11;9:e1459. doi: 10.7717/peerj-cs.1459 (PMC10403181; doi:10.7717/peerj-cs.1459)
Supplement: Supplemental Information 1 [file peerj-cs-09-1459-s001.docx]

| **Method** | **Characteristics** | **Limitations** |
| --- | --- | --- |
| Correlated Topic Model  (CTM) | Uses a normal logistic distribution to create relations among topics  Allows the occurrences of words in other topics and topic graphs | Requires lots of calculation  Results in lots of general words inside the topics |
| Dirichlet Multinomial Regression (DMR) | Uses Gibbs sampling  Provides inferences about hidden variables | Tendency to underestimate abundant features and overestimate marginal features  Results in a larger number of variables to sample and a more complicated sampling distribution (low efficiency) |
| Hierarchical LDA (HLDA) | Discovers topics within a corpus in a hierarchical way  Puts abstract terms at the base of the hierarchy  Locates detailed and specific terms near the leaves of the hierarchy | Ignores the lexical co-occurrence  Poor consideration for word dependencies  The more the hierarchy levels, the slower the performance (long execution time) |
| Latent Dirichlet Allocation (LDA) | Requires manual removal of stopwords  Previous studies have found that representation of the relationships among topics is out of LDA's scope | Inability to model relations among topics  The number of topics (K) must be known  Failure in the face of a large number of vocabularies |
| Pseudo-document based Topic Model (PTM) | Analyses topics without using auxiliary contextual information  Assumes each short text relates to only a single pseudo document  Avoids overfitting when the training corpus is in a relative shortage | Cannot be applied directly to raw input data (needs to use some heuristic methods to enrich the input data)  Lacks a quantitative relationship among words  Can generate some high-frequency but topic-irrelevant words  Cannot deal with extremely sparse and noisy data |
| Supervised Latent Dirichlet Allocation (sLDA) | Assigns a label on each training document (in distinction from the LDA model)  Offers improved predictions over regressions on words alone  Applicable, besides text, on social networks image classification | Requires marking documents with a response variable  Cannot be used for multi-class classification problems  Labour-intensive and expensive to apply it on a large dataset due to the labelling process |
