# Supplementary figures and images for: Web content topic modeling using LDA and HTML tags
Source: PeerJ Comput Sci. 2023 Jul 11;9:e1459. doi: 10.7717/peerj-cs.1459 (PMC10403181; doi:10.7717/peerj-cs.1459)

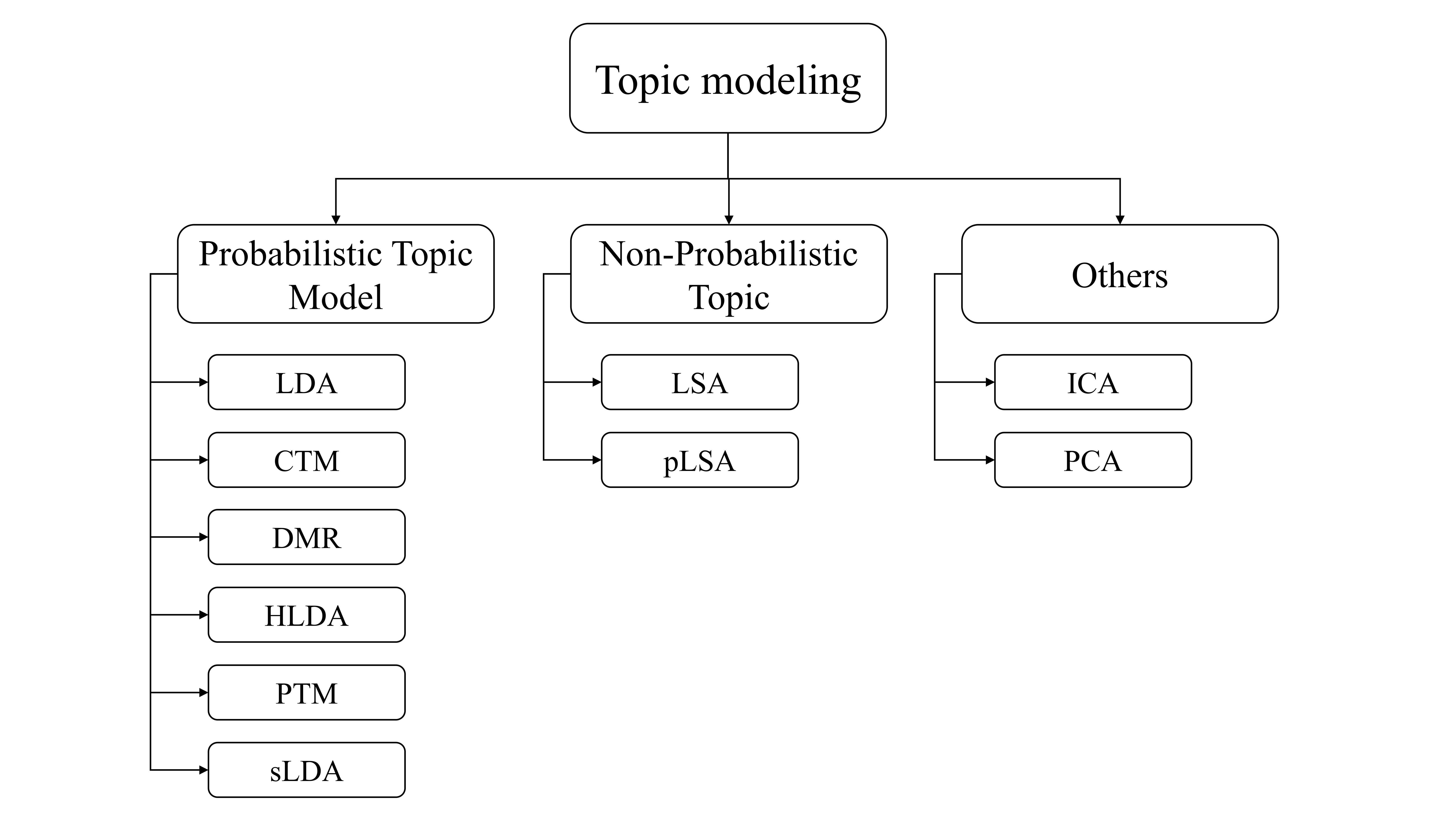

Supplement: Supplemental Information 2 [file peerj-cs-09-1459-s002.png]

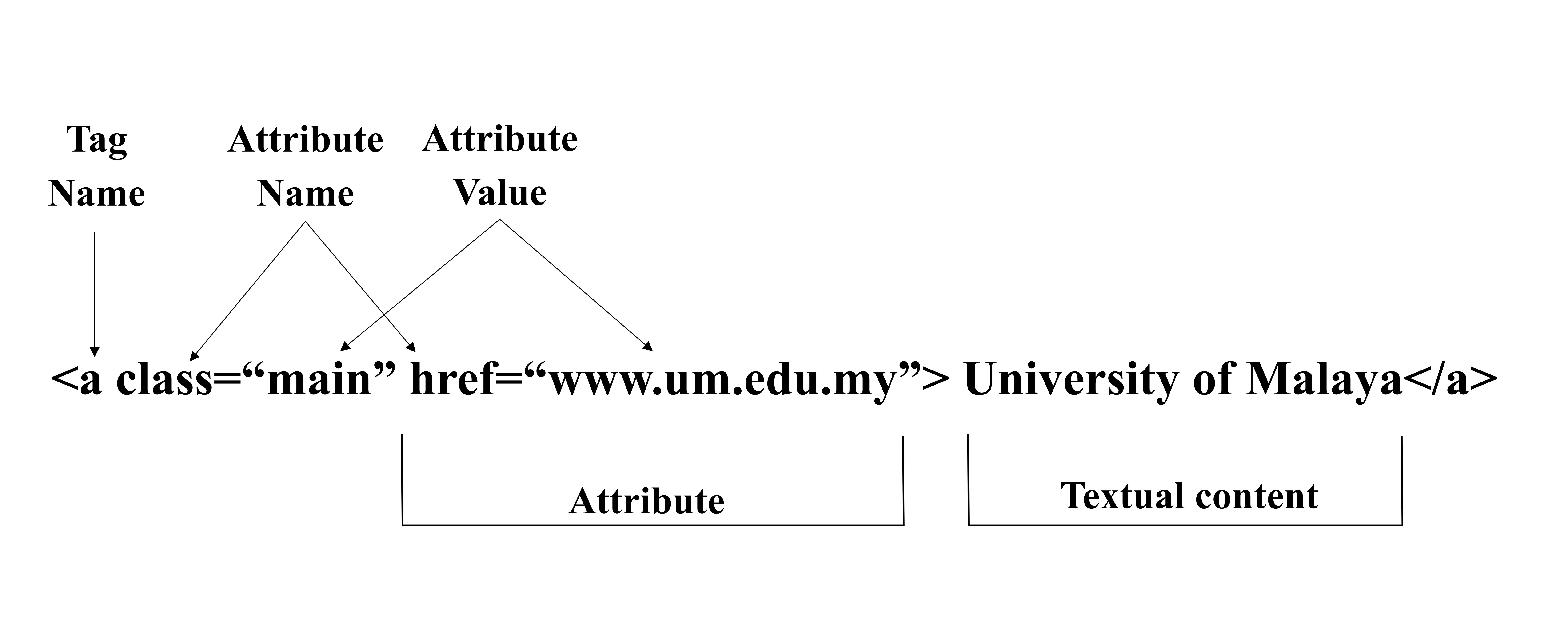

Supplement: Supplemental Information 3 [file peerj-cs-09-1459-s003.png]

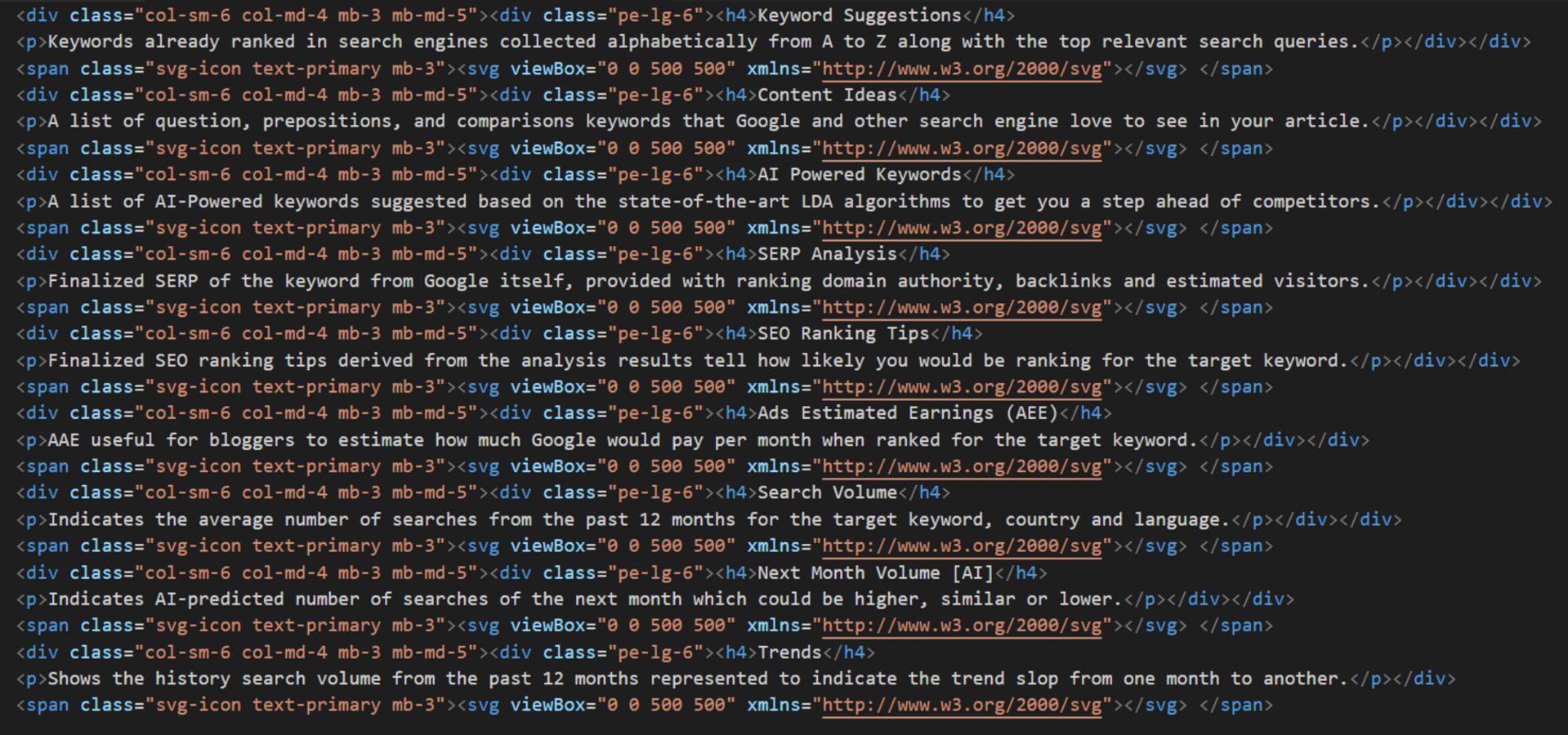

Supplement: Supplemental Information 4 [file peerj-cs-09-1459-s004.png]

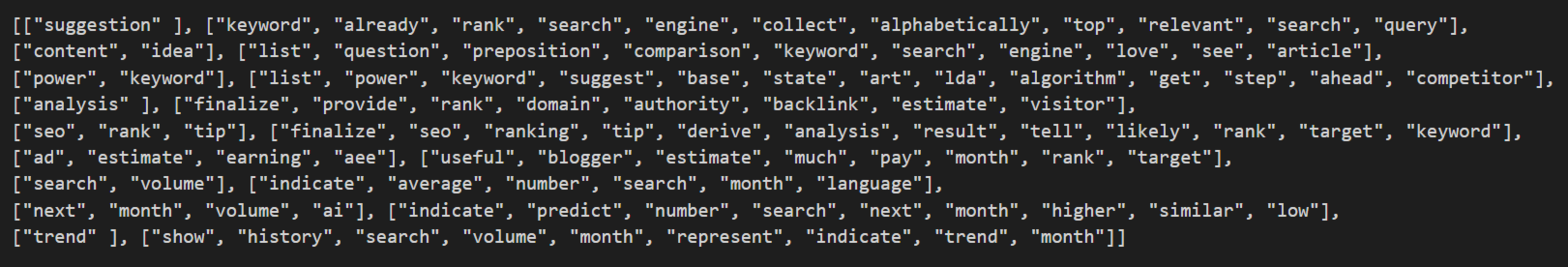

Supplement: Supplemental Information 5 [file peerj-cs-09-1459-s005.png]

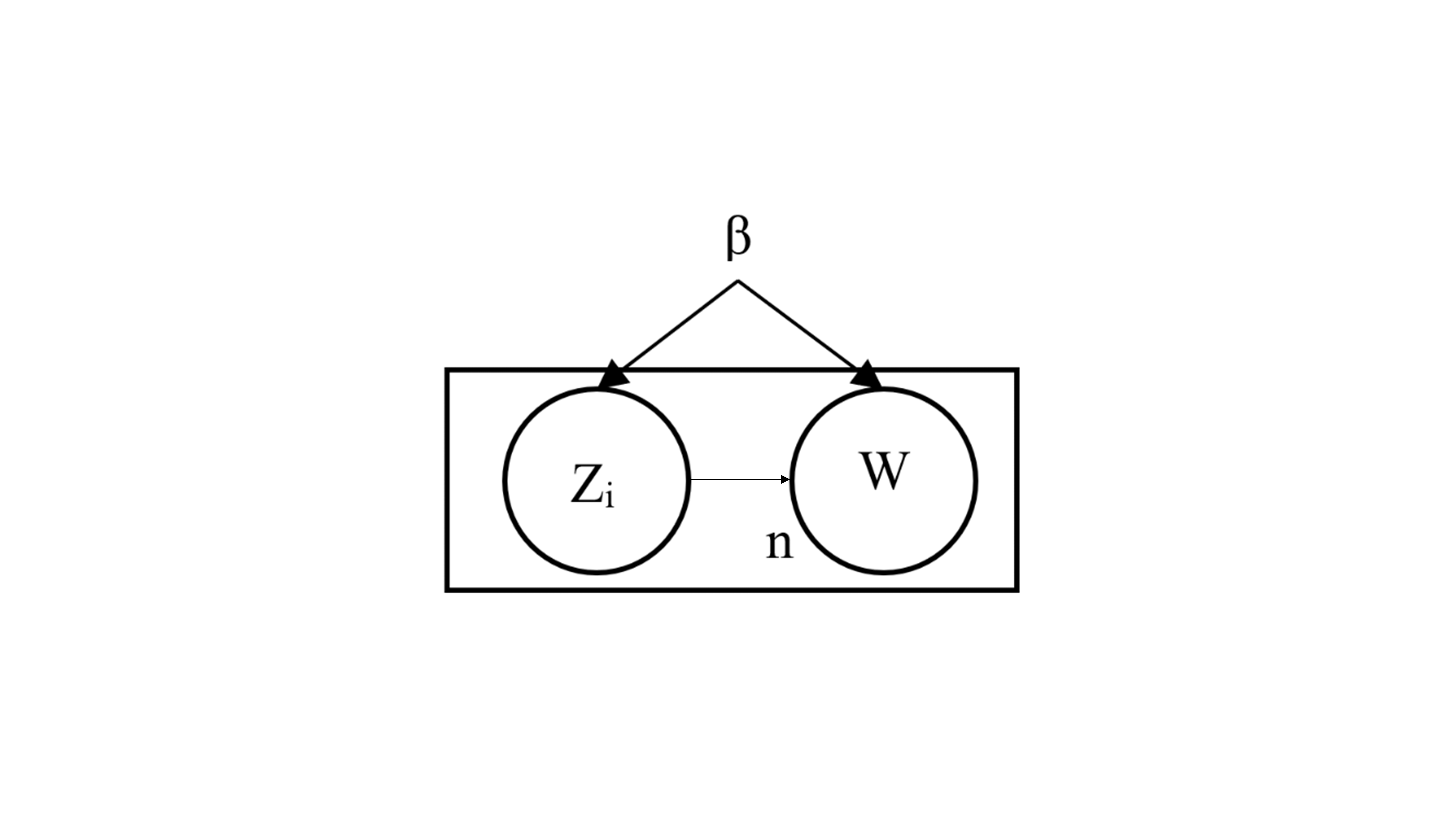

Supplement: Supplemental Information 6 [file peerj-cs-09-1459-s006.png]

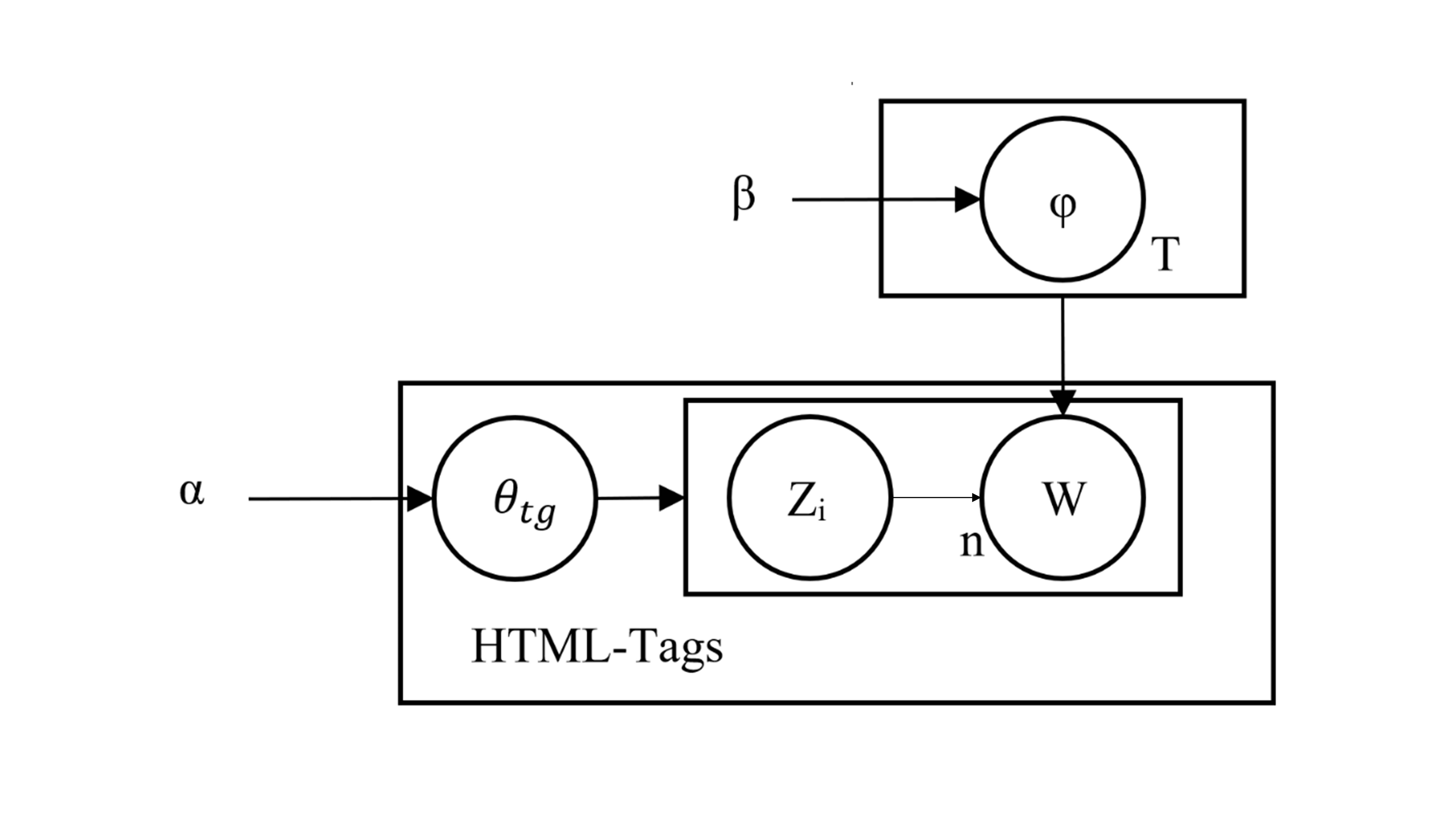

Supplement: Supplemental Information 7 [file peerj-cs-09-1459-s007.png]

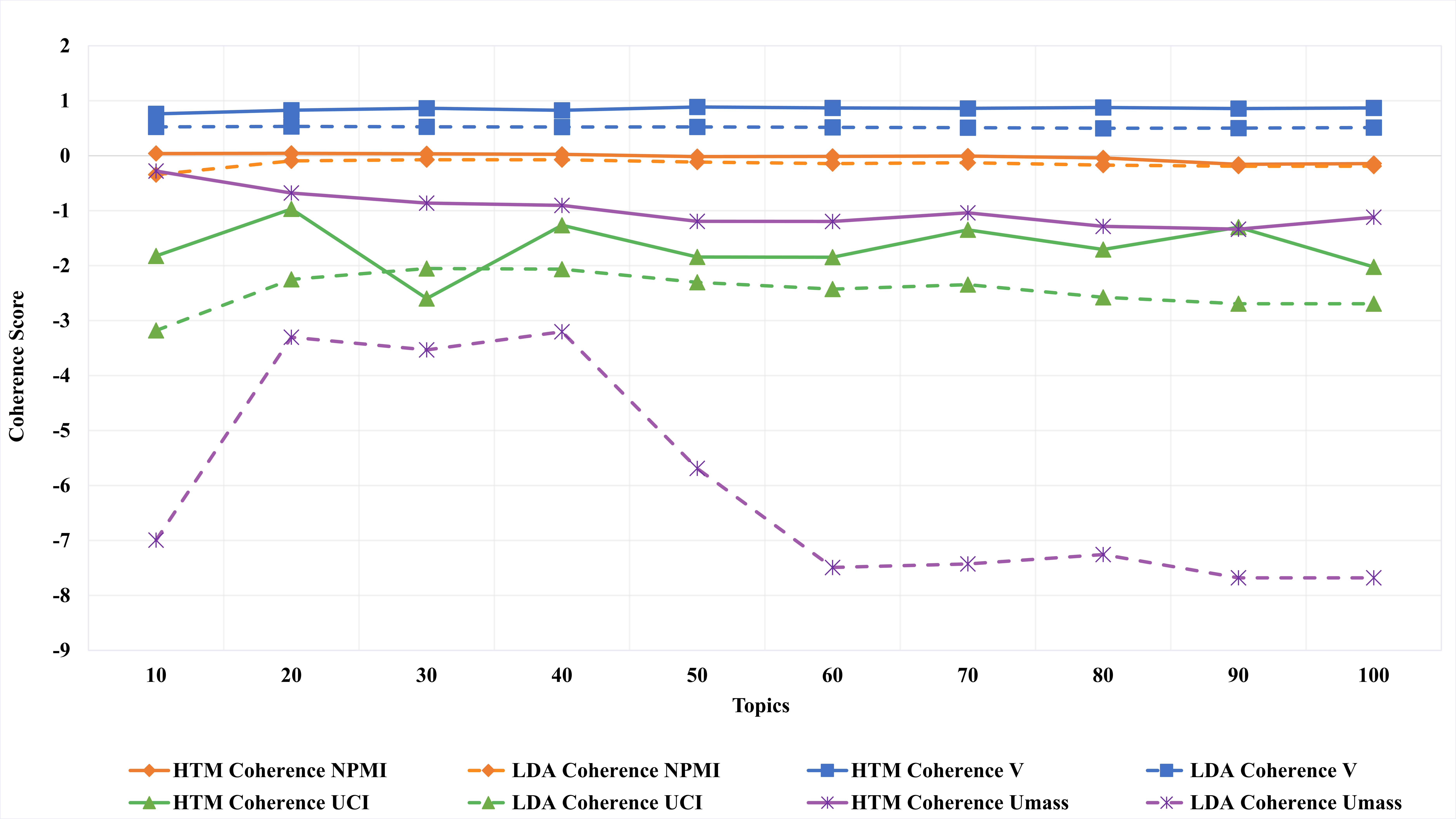

Supplement: Supplemental Information 8 [file peerj-cs-09-1459-s008.jpg]
